# Combined treatments with microballoon catheters and multiple cryoablation probes for shoulder-subclavian soft tissue hemangiopericytoma: A case report

**DOI:** 10.1016/j.radcr.2023.06.063

**Published:** 2023-07-17

**Authors:** Renato Argirò, Giorgio Ciccarese, Leonardo Vattermoli, Sara Crociati, Vincenzo Iannibelli, Valentina Forte, Daniele Morosetti, Roberto Floris

**Affiliations:** a Interventional Radiology Unit, Department of Biomedicine and Prevention, University of Rome “Tor Vergata”, Viale Oxford 81, Rome, 00133, Italy; b Diagnostic Imaging Unit, Department of Biomedicine and Prevention, University of Rome Tor Vergata, Viale Oxford 81, Rome, 00133, Italy; c Neuroradiology Unit, Department of Biomedicine and Prevention, University of Rome “Tor Vergata”, Viale Oxford 81, Rome, 00133, Italy; d Neurology Unit, Department of Neurology, University of Rome Tor Vergata, Viale Oxford 81, Rome, 00133, Italy

**Keywords:** Microballoon intervention, Cryoablation, Combined treatment, Hemangiopericytoma, Fluoroscopy, MRI, CT-scan

## Abstract

We describe a case of a 65-year-old woman affected by hemangiopericytoma/solitary fibrous tumor of the right shoulder—subclavian region. Hemangiopericytoma/solitary fibrous tumor is a rare tumor of uncertain malignancy. She reports shoulder pain and inability to abduct the arm and elevate the shoulder. Imaging showed erosion of the scapula. The patient underwent 5 sessions of “on demand” embolization in the previous 2 years scheduled for recurrence of symptoms-swelling of tissues. Further 2 treatments were achieved through embolization via 2 different microballoon catheter combined with percutaneous cryoablation with 5 probes. Images after the treatment demonstrate a marked reduction in the hypervascularized area and an increase in the necrosis area. So, this combined treatment is safety and reproducible also in extrahepatic tissue.

## Introduction

Hemangiopericytoma (HPC)/solitary fibrous tumor is a very rare tumor of uncertain malignancy [1]. In 1942, Stout and Murray first characterized these neoplasms as “vascular tumors resulting from Zimmerman's pericytes” and until now HPCs and solitary fibrous soft tissue tumors are considered to be characteristic of the same entity in the soft tissue fascicle.

The incidence of the head and neck is less than 20%, mainly in adults [2], [3], [4].

Both embolization and ablation with different types of energy have not already been described for percutaneous emangiopericytoma treatment.

Here, we describe a case of percutaneous combined treatment of right shoulder—subclavian region hemangioperycitoma with contemporary embolization through 2 differentx microballoon catheter (Occlusafe™ Temporary Occlusion Balloon Catheter—Terumo) combined with five 14 G cryoablation probes (IceSphere™ and IceForce™-Boston Scientific).

## Case report

We present a case of a 65-year-old woman affected by HPC/solitary fibrous tumor of the right cervical and supraclavicular-shoulder. The mass was small and soft on palpation; the skin over the tumor was intact and normal. She had neither tumor-related disease nor any other type of pathology in her medical history. Histologic analysis was achieved after surgical biopsy in 2018; biopsy was complicated by hemorrhage requiring urgent transarterial embolization with coils and Gelatin sponge (Spongostan®).

Patient was asymptomatic till 2020 when referred progressive swelling of the perihumeral-scapolar tissue with loose of adduction-elevation of shoulder joint.

**Fig. 1:**
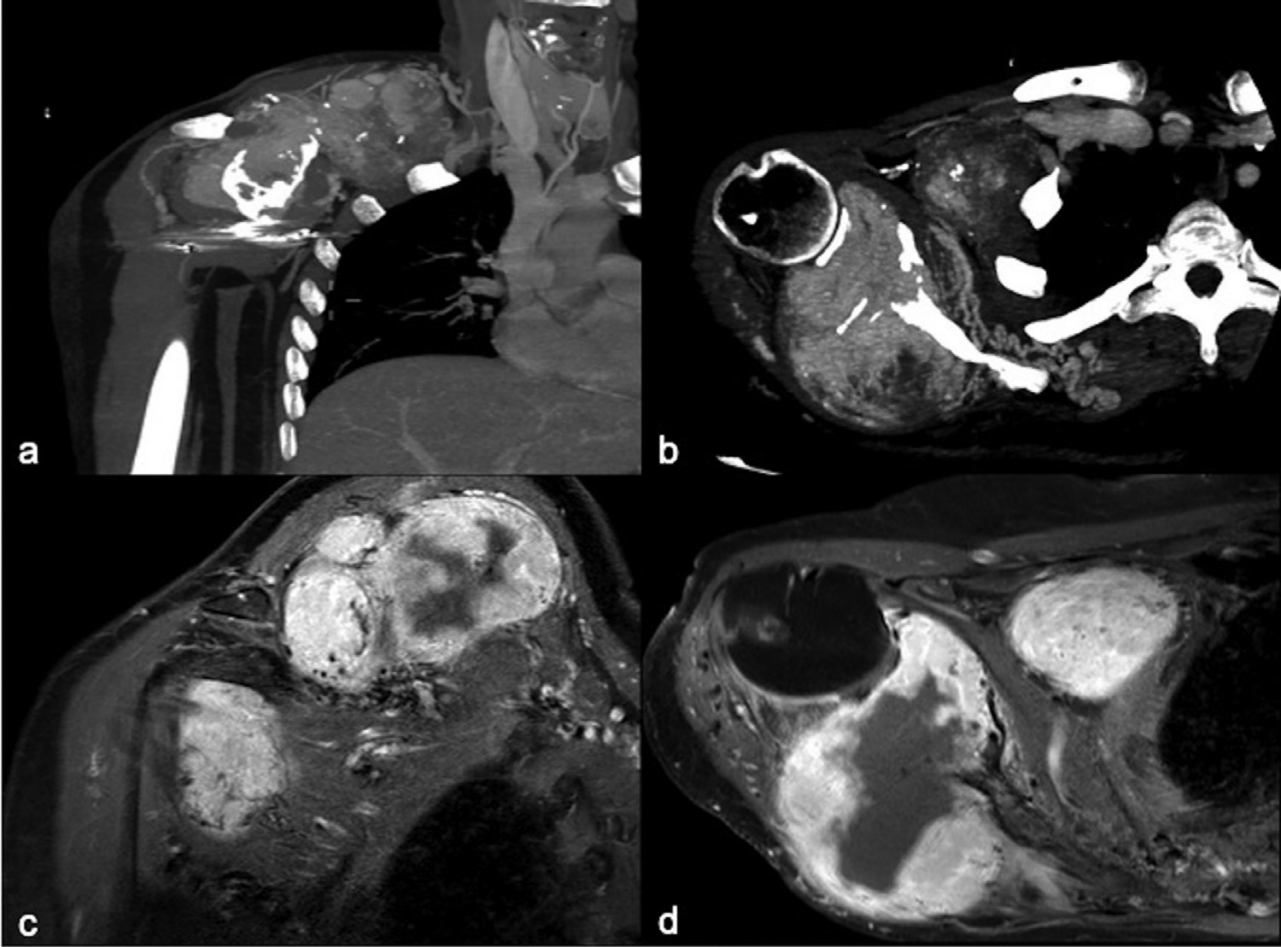
(A, B) Pretreatment Maximum Intensity Projection postcontrast venous coronal and axial CT before 2 combined treatment depicting the hypervascular hemangiopericytoma with erosion of the scapula. (C) Postcontrast coronal MRI after the 2 combined treatment focused in depicting the necrotic area in the medial portion of the lesion. (D) Postcontrast axial MRI after 2 combined treatment focused in depicting the necrotic area in the lateral portion of the lesion.

**Fig. 2:**
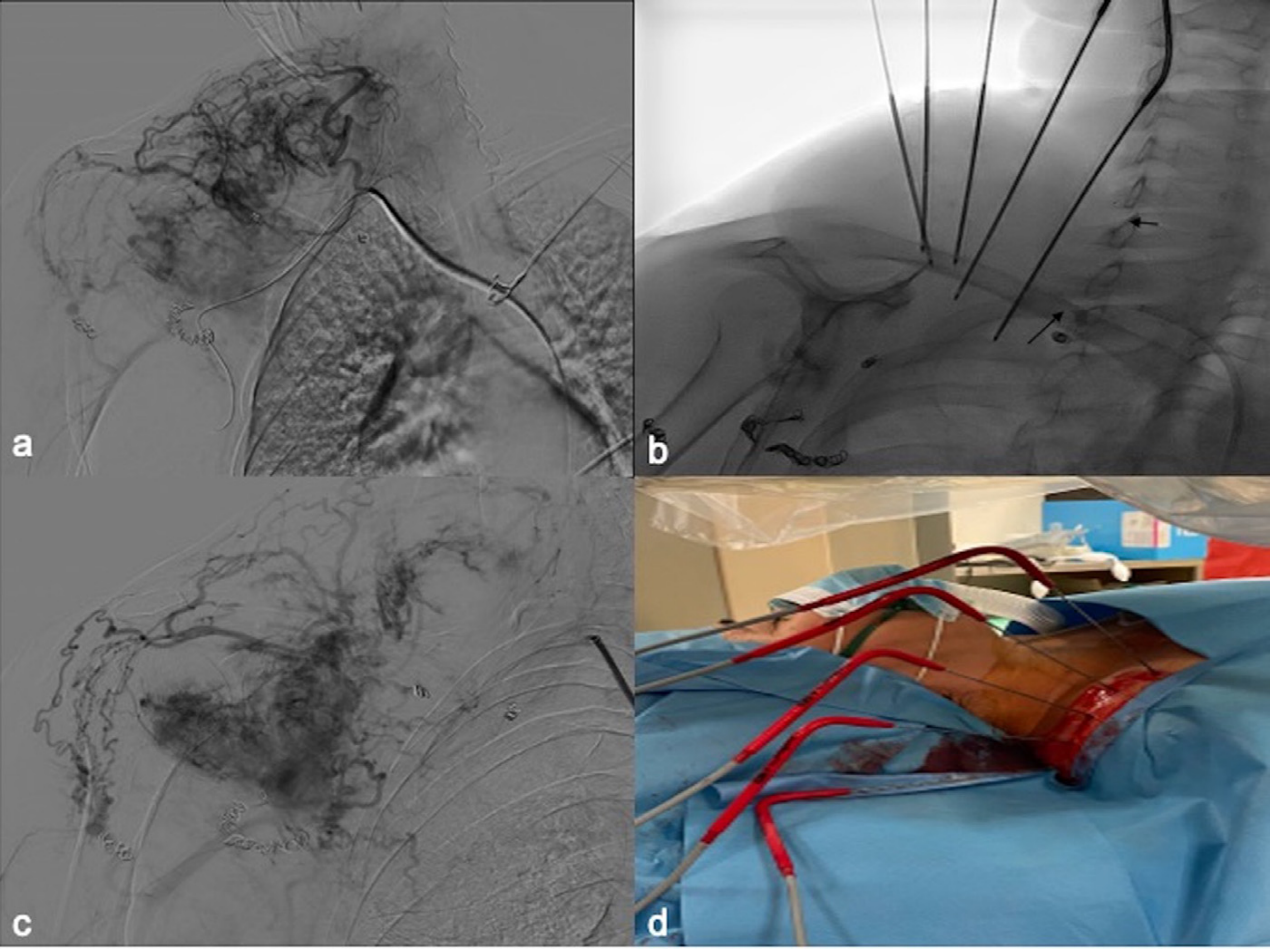
First combined treatment of the medial portion of the lesion. (A) Preprocedure angiography from 6 Fr sheet from right subclavian artery demonstrates hypervascular lesion in periscapular-subclavian region with multiple feeder arising from subclavian and axillary arteries. (B) Fluoroscopic image depicts 5 cryobrobes and 2 microballoon catheters positioned in the 2 main feeders (arrows). (C) Postprocedure angiography demonstrating marked reduction of vascularization in the treated portion of the tumor. (D) Picture of the 5 cryoprobes.

**Fig. 3:**
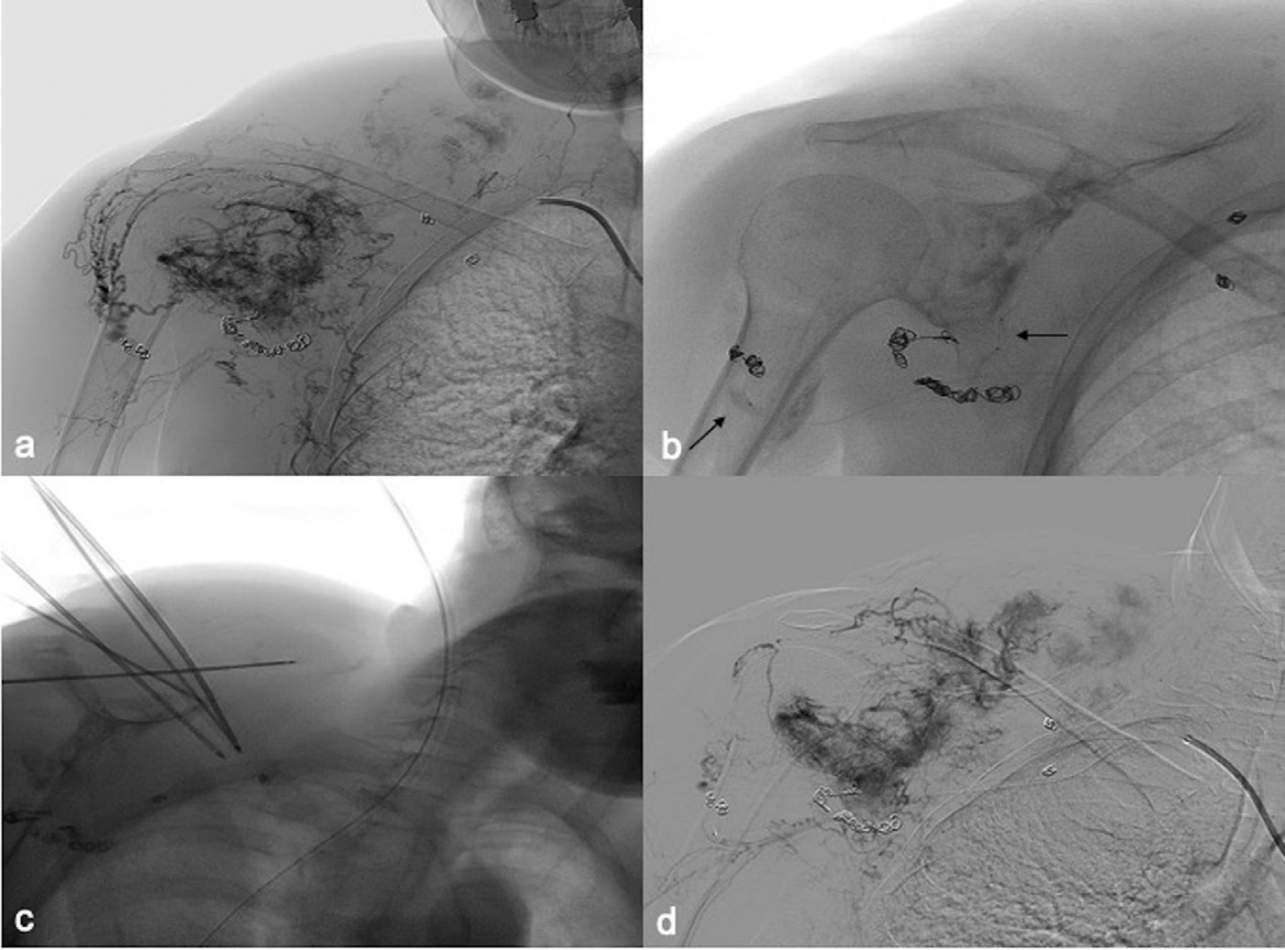
Second combined treatment of the lateral portion of the lesion. (A). Preprocedure angiography from 6 Fr sheet from right subclavian artery demonstrates hypervascular lesion in periscapular-subclavian region with multiple feeder arising from subclavian and axillary arteries; angiography demonstrates reduction of vascularization in the previous treated portion. (B) Fluoroscopic image depicting 2 microballoon catheters positioned in the 2 main feeders (arrows). (C). Fluoroscopic image shows 5 cryoprobes positioned in the lateral portion of the lesion. (D) Postprocedure angiography demonstrating marked reduction of vascularization in the treated portion of the tumor.

Patient underwent CT and MRI with evidence of a near total (14 × 13 × 8 cm) capsulated mass with compression of locoregional structure (brachial plexus) and sign of erosion of scapula (Figs. 1A,B). Imaging revealed the presence of hypertrophic, tortuosus arteries network arising from subclavian and axillary artery (Figs. 2A and 3A).

Radical surgical treatment was proposed but due to possibility of loose of arm function and shoulder major amputation, the patient refused this option.

The patient reported persistent pain in the right shoulder because the growing tumor came into conflict with the shoulder joint causing inability to move. In addition, the growing mass caused a large swelling in the neck and right shoulder region.

Patient was therefore treated with multiple session of embolization (5 in total) in a 2-year period with “on demand” schedulation based on to recurrence of symptoms-swelling of tissues. Previous embolizations were performed in a superselective catheterization of major feeders via femoral access, 6 Fr guide catheter and a 2.4 Fr or 1.9 Fr microcatheters with infusion of microsphere of different calipers (in every treatment from 100 to 900 micron).

Treatments were interspersed by a period of 4-6 months with good technical success and reduction of symptoms and implementation of shoulder-joint mobility.

Due to recurrence of symptoms, 2 other interventions were scheduled 6 weeks apart in order to treat first the medial part and then the lateral part of the lesion. Therefore, in every session, combined treatment with contemporary positioning of 2 microballoon catheter and 5 different cryoprobes was achieved. A superselective catheterization of the 2 main feeders of every portion of the lesion was performed and microballoon catheter was inflated to occlude the flow and obtain balloon-occluded arterial stump pressure (Figs. 2B and 3B). Therefore, under US guidance, 5 different 14 G cryoprobes were 3-dimensionally positioned and 2 cycle of freezing-thawing was carried out (Figs. 2B,D and 3C) [5]. Before cryoprobes retrieve heat track ablation was performed to reduce bleeding possibility. Subsequently thought the 2 microballoon catheter embolization with 75-200-400-600 micron hydropearl (HYDROPEARL™ Microspheres - Terumo) was performed till hemostasis was observed (Figs. 2C and 3D). No major-minor complication occurred.

## Discussion

Given their rarity, optimal therapeutic strategies for these tumors have not been well established and surgery to date remains the only effective treatment capable of providing significant survival benefits in patients with HPC, while the clinical utility of adjuvant chemotherapy or radiotherapy is limited [1].

Adjuvant radiation therapy and radiosurgery may be helpful in cases of incomplete resection. Stereotactic radiosurgery has been evaluated as an adjuvant treatment after surgical resection, as well as a primary treatment for new or recurrent HPC. It can deliver a higher dose to the target with a sharper drop into the surrounding tissue.

As angiogenesis is one of the key features leading to tumor invasion and metastasis, these pathways may be used as potential therapeutic targets, and they represent the most promising research area for future studies [6].

Balloon microcatheter intervention had been described in liver chemoembolization for Hepatocellular Carcinoma [7,8]. Reported advantages of this technique are reversal of the flow of collateral arteries, reduction in the number of superselective catheterizations, and improvement of results on larger nodules in a single session.

In recent years, combined treatment with microwave antenna and Occlusafe had been proposed as an innovative technique in treatment of >3cm liver malignancies with promising results [9].

Occluded balloon procedures have been shown to be safe, with an equivalent adverse event rate to nonocclusive procedures. Furthermore, it has been shown that they are able to positively influence the oncological outcome of treated patients, regardless of the embolic agent used.

To date, the only reported complication directly related to microballoon use has been vascular dilatation at the balloon inflation site. In all reported cases, this event was asymptomatic and was revealed during routine follow-up with contrast-enhanced CT and required no further treatment [7].

These catheters can be difficult to place in case of excessive vessel tortuosity, small vessels, and anatomical complexity [10].

We prefer to use cryoablation as ablation technique due to the proximity of nervous and musculoskeletal tissue and also to increase pain management.

This decision was taken because the combination of the 2 methods allows a larger ablation zone and greater precision, compared to other methods such as radiofrequency ablation and other thermal modalities [11].

The minimally invasive nature of the treatment helps reduce the risk of surgical complications such as infection, bleeding, and scarring. Recovery times are faster and postoperative pain is reduced. This means less impact on quality of life and faster return to normal work activities. The use of local anesthesia reduces the risks associated with general anesthesia and allows patients to remain conscious during the procedure. Furthermore, it is associated with a shorter procedure duration compared to open surgery and reduced damage to surrounding tissues, allowing the target area to be accurately reached. Therefore, HPC are hypervascular tumors prone to extensive bleeding, so it is often impossible to perform a complete resection.

In our case, the use of the microballoon catheter, despite its application in a different district than the hepatic one, thanks to collateral flow, demonstrates an increase of the embolization territory with single positioning of the microcatheter; indeed, in the previous embolization sessions, multiple superselective microcatheterisms were necessary to achieve an adequate embolization of the tissue. This leaded to a reduction of procedural times.

In addition, as results of the previous treatments, a reduction in the number of catheterizable feeders was observable and therefore through a contemporary double-balloon microcatheter positioning, an increase in the embolization area was achieved.

Furthermore, in our case, the combined procedure of cryoablation treatment during arterial stop-flow made it possible a “debulking” before the embolization, resulting in an increase in the volume of the treated area (Figs. 1C, D).

The MRI performed 1 month after the 2 combined treatments showed a mild reduction in size of the lesion and marked reduction in the hypervascularized area and an increase in the necrosis area (7.5 × 2.5 × 2.5 cm) with complete regression of symptoms.

## Conclusions

Employment of microballoon catheter in extrahepatic oncological treatment has not been already described. To our knowledge, this is a first case treated with the combination of these 2 materials. In this case, the results we obtained demonstrate its feasibility and safety profile. Further, future studies are needed to achieve standardization of this combined technique in extrahepatic disease.

## Ethical approval

All procedures performed in studies involving human participants were in accordance with the ethical standards of the institutional and/or national research committee and with the 1964 Helsinki declaration and its later amendments or comparable ethical standards.

## Patient consent

To whom it may concern, hereby I confirmed that I have obtained the written informed patient consent for the publication of this case report.
